# Cardiovascular risk factor distribution and subjective risk estimation in urban women – The BEFRI Study: a randomized cross-sectional study

**DOI:** 10.1186/s12916-015-0304-9

**Published:** 2015-03-16

**Authors:** Sabine Oertelt-Prigione, Ute Seeland, Friederike Kendel, Mirjam Rücke, Agnes Flöel, Wolfgang Gaissmaier, Christine Heim, Renate Schnabel, Verena Stangl, Vera Regitz-Zagrosek

**Affiliations:** Institute of Gender in Medicine, Charité – Universitätsmedizin, Hessische Str. 3-4, 10115 Berlin, Germany; Deutsches Zentrum für Herz- Kreislaufforschung (DZHK) Partner Site, Berlin, Germany; Institute of Medical Psychology, Charité – Universitätsmedizin, Luisenstr. 57, 10117 Berlin, Germany; Department of Neurology, Charité – Universitätsmedizin, Charitéplatz 1, 10117 Berlin, Germany; Department of Psychology, University of Konstanz, Universitätsstr. 10, 78457 Konstanz, Germany; University Heart Center, University Medical Center, Hamburg-Eppendorf, Martinistraße 52, 20246 Hamburg, Germany; Division of Cardiology and Angiology, Charité – Universitätsmedizin, Charitéplatz 1, 10117 Berlin, Germany; Center for Cardiovascular Research, Charité – Universitätsmedizin, Hessische Str. 3/4, 10115 Berlin, Germany

**Keywords:** Awareness, Cardiovascular disease, Health literacy, Prevention, Risk factors, Women

## Abstract

**Background:**

Awareness represents a major modulator for the uptake of preventive measures and healthy life-style choices. Women underestimate the role of cardiovascular diseases as causes of mortality, yet little information is available about their subjective risk awareness.

**Methods:**

The Berlin Female Risk Evaluation (BEFRI) study included a randomized urban female sample aged 25–74 years, in which 1,066 women completed standardized questionnaires and attended an extensive clinical examination. Subjective estimation was measured by a 3-point Likert scale question asking about subjective perception of absolute cardiovascular risk with a 10 year outlook to be matched to the cardiovascular risk estimate according to the Framingham score for women.

**Results:**

An expected linear increase with age was observed for hypertension, hyperlipidemia, obesity, and vascular compliance measured by pulse pressure. Knowledge about optimal values of selected cardiovascular risk factor indicators increased with age, but not the perception of the importance of age itself. Only 41.35% of all the participants correctly classified their own cardiovascular risk, while 48.65% underestimated it, and age resulted as the most significant predictor for this subjective underestimation (OR = 3.5 for age >50 years compared to <50, 95% CI = 2.6–4.8, *P* <0.0001). Therefore, although socioeconomic factors such as joblessness (OR = 1.9, 95% CI = 1.4–2.6, *P* <0.0001) and combinations of other social risk factors (low income, limited education, simple job, living alone, having children, statutory health coverage only; OR = 1.5, 95% CI = 1.1–2.1, *P* = 0.009) also significantly influenced self-awareness, age appeared as the strongest predictor of risk underestimation and at the same time the least perceived cardiovascular risk factor.

**Conclusions:**

Less than half of the women in our study population correctly estimated their cardiovascular risk. The study identifies age as the strongest predictor of risk underestimation in urban women and at the same time as the least subjectively perceived cardiovascular risk factor. Although age itself cannot be modified, our data highlights the need for more explicit risk counseling and information campaigns about the cardiovascular relevance of aging while focusing on measures to control coexisting modifiable risk factors.

**Electronic supplementary material:**

The online version of this article (doi:10.1186/s12916-015-0304-9) contains supplementary material, which is available to authorized users.

## Background

Cardiovascular diseases represent the primary cause of mortality for women worldwide and their relevance is projected to increase significantly in upcoming years. These diseases are not only major causes of mortality, but also increasingly relevant as causes of long-term disability given the progressive improvement of medical care. In fact, the World Health Organization predicts that, in 2030, the disability related to ischemic heart disease and cerebrovascular disease will amount to 9.8% of all disability-adjusted life years worldwide [1]. Many large clinical studies have proven the partial preventability of cardiovascular disease with lifestyle measures, such as smoking cessation [2], healthy eating [3,4], physical activity [5], and weight [6] and stress management [7]. Active risk reduction becomes increasingly relevant as the individual ages; since ageing represents one of the most relevant uncontrollable risk factors the general population is exposed to [8]. Even though the importance of cardiovascular disease for women is well documented, subjective awareness is limited [9]. Information campaigns such as the American Heart Association’s “go red for women” have proven partially effective in raising awareness in the female population in the United States [10], yet similar actions have not been implemented in Europe. Small studies in the German female population provide evidence for an analogous underestimation of cardiovascular risk in favor of breast cancer as the primary cause of mortality for women [11].

While these studies document the underestimation of the objective role of cardiovascular diseases as causes of mortality, the role of underestimation of one’s subjective cardiovascular risk has never been documented in the female population. With regards to targeted prevention, however, self-perception of risk and risk awareness represent indisputable steps for uptake. In fact, underestimation of risk has not only been linked to an increase in risky behavior [12], it is also believed to be associated with the limited uptake of preventive offers [13]. Thus, improvement of self-awareness might represent an essential step to increase commitment to prevention and healthy life-style choices.

This study was designed to identify predictors of incorrect subjective risk estimation in the general female urban population in Germany, with the aim to pinpoint susceptive groups for future targeted health literacy interventions.

## Methods

### Study population

The Berlin Female Risk Evaluation (BEFRI) study was designed to appropriately represent the city’s population (Additional file 1: Table S1). The city of Berlin is divided into 12 boroughs with different social structure and different population density. We aimed to obtain a study population of 1,000 women that could appropriately represent the city’s population. Thus, in accordance with response rates of about 30 to 40% in German studies with more complex recruiting strategies [14], we envisioned a response rate between 20 and 30% for our strategy and invited 3,600 women to participate. Women were randomly selected by the city census (Einwohnermeldeamt). Inclusion criteria were age between 25 and 74 years, represented in five equal age strata, and proportional representation of the boroughs inhabitant density, i.e., densely populated boroughs contributed more participants than scarcely populated ones.

Randomized women were sent a single invitation letter and one reminder by regular mail describing the study’s purpose and the exams to be conducted in case of acceptance. If women wanted to participate or obtain more information before consenting, they were advised to notify the study coordination center by phone, mail, or e-mail. All women who expressed the intention to participate (n = 1,199) were sent the questionnaire, consent forms, and further information materials by mail and an appointment was scheduled, if requested. Of these 1,066 (88.9%) agreed to participate; 3 of these women were excluded from analysis because they had reached age 75 by the time they attended the appointment and 1 due to major literacy issues demonstrated upon clinical interview. We compared social indicators within our population with the census data of the city of Berlin (reference sample) and cardiovascular risk factor prevalence with German national data (Additional file 1: Table S1) confirming a valid representation of the city’s population structure and risk factor distribution. Once women agreed to participate, appointments were scheduled either at Charité hospital or externally at the borough level based on the participants’ preference.

### Examinations

At the appointment, written informed consent was obtained, a standardized medical history was taken, and clinical examinations were performed. Data was collected in paper form and recorded using an Access (Microsoft Corp., Seattle, WA, USA) database for management during the recruitment period. All patient data was pseudonymized at the collection site using serial numbers. Participants were first asked if any items within the questionnaires needed clarification and, if needed, further information was given for unclear questions. Medical history focusing on the presence of common risk factors, history of previous diseases, and specific cardiovascular symptoms and manifestations was taken. Previous and current hormone therapy, as well as current medication use was also recorded. Clinical examinations were then performed. All women were weighed lightly clothed on a standard Bosch scale (Axxence Spirit, Bosch GmbH, Stuttgart, Germany) to the nearest 0.1 kg, and measured to the nearest 1 mm using a stadiometer (Seca 216, Hamburg, Germany) whilst standing completely erect without shoes. Waist and hip circumference were determined with a non-stretchable standard tape measure placed straight at the narrowest level at the waist between the costal margin and iliac crest and hip circumference at the largest level across the buttocks. Women were then asked to lie down for electrocardiogram (ECG), blood pressure, and arterial stiffness measurements. A 12-lead ECG was performed following the standardized procedure (AT-10plus, Schiller, Baar, Switzerland). Measurement of blood pressure on both arms and legs and of vascular stiffness parameters were obtained after at least 10 minutes rest with an Arteriograph device (TensioMed Ltd., Budapest, Hungary). Venipuncture was performed lying down or in an upright position based on the participant’s preference with a Monovette (Sarstedt AG, Nümbrecht, Germany) system. Blood was collected in ethylenediaminetetraacetic acid and citrate tubes and placed immediately into a refrigerator at 4°C until collection. All samples were transferred to the processing lab on dried ice or cool packs the same day and processed within 24 hours from collection. Full blood counts, renal function, lipid metabolism, glycated haemoglobin, b-type natriuretic peptide, and sexual hormones were determined at an external lab (Hospital Laborverbund Berlin-Brandenburg, Bernau, Germany). Diagnoses of hypertension and diabetes were based on internationally accepted criteria [15,16]. Autoimmune diseases were classified according to the American College of Rheumatology guidelines. Upon completion of the examination, participants obtained a reimbursement of 50 Euros. In order to minimize selection bias, information about the compensation was only given once women had agreed to participate. The institutional ethics committee approved the study (Ethikkommission, internal identification number EA2/116/12) and all research conforms to the Declaration of Helsinki.

### Questionnaires

All participants were sent a specifically designed questionnaire to assess several domains: sociodemography, clinical and family history, gynecological anamnesis, physical fitness, prevention history, and psychosocial dimensions. Sociodemographic variables were elicited using standard questions from the German Health Survey ([17] with permission from Robert Koch Institute) or slight modifications thereof, as well as investigator-designed questions, if needed. Clinical and family history assessment contained detailed questions about a large number of cardiovascular conditions in the family and partner. Participant risk factors, as well as presence of myocardial infarction, stroke, thromboembolic events, vascular events, or sudden death were documented. Gynecological history mostly focused on physiological events and pregnancy complications, as well as previous or current hormone therapy. Physical work capacity was determined using the Duke Activity Status Index score [18]. Subjective risk was determined by a direct question about subjective estimation of absolute cardiovascular risk with a 10-year outlook using a 3-point Likert scale answer option (low, medium, high). The question was embedded in a set of questions about risk perception, introduced by a description of the nature of cardiovascular disease and its potential outcomes, such as myocardial infarction or stroke. Prevention history and expectations were elicited with specifically designed questions. General health perception and quality of life was elicited using the SF-12, the short form of the SF-36 questionnaire [19]. Social support, depression, and exposure to critical life events were tested using standardized measures [20]. The full questionnaire was piloted in a set of 50 women of different age and socioeconomic and educational level to test feasibility, duration, and comprehension. Single questions were adapted based on piloting results. Average completion time was 75 minutes.

### Statistical analysis

The main hypothesis to be tested within this study was the presence of a statistically significant subjective underestimation of cardiovascular risk in the general population. The secondary aim was the identification of modifying factors for incorrect estimation of cardiovascular risk. Descriptive statistics were computed to identify risk factor distributions within age groups and to allow a comparison of the study population with the mean demographics of the urban reference sample. Subjective estimation was correlated with the three-tiered Framingham global cardiovascular disease risk score for women [21] to define correctness of estimation. This score was selected due to its high validation, compared to QRISK [22] or the Reynolds Risk score [23], which could have been otherwise valid alternatives, and its ability to represent the breadth of cardiovascular disease, rather than focusing on solely coronary heart disease and its acute consequences, which would underestimate risk in women. Furthermore, it will also allow for comparison with a male study population in the future. Univariate analyses defining the association of single risk factors with over- or underestimation using correct estimation as the baseline was then performed. Tests for trend were performed using a non-parametric Cuzick test for trend, an extension of the Wilcoxon rank-sum test. Two-tailed analyses were conducted and a *P* value <0.05 was considered statistically significant. Since missing rates for demographic and laboratory parameters were <3% in the whole dataset, no imputation was calculated for missing data. Following this, multivariate logistic models were fitted with under- or overestimation as outcome variables. Different strategies were employed to ascertain model robustness. A model using all variables from the univariate analysis was fitted and reduced progressively by elimination of non-significant (*P* >0.1) explanatory variables to avoid overfitting. Interaction terms among significant variables in the univariate analyses were included, but proved non-significant. Likelihood ratio tests were performed at each step to confirm redundancy of the eliminated variables. This was conducted until no further reduction was possible. Automated forward and backward regression models were also calculated and confirmed the final model. Model misspecification was assessed by the Wald test and goodness-of-fit by the Hosmer-Lemeshow test. All statistical analyses were performed using STATA Version 13 (STATA Corp, College Station, TX, USA).

## Results

### Sociodemographic characteristics of the study population

The complete study population (n = 1,062) was divided into five equally-powered age groups (Table 1). Sociodemographic descriptors were distributed in accordance with the reference urban population of the city of Berlin (Additional file 1: Table S1); 46.1% of the women in the study population displayed lower educational status with older women more frequently reporting less years of formal education. In fact, the youngest age stratum was the only one diverging from the reference sample, with a higher representation of well-educated women. Further, 61.7% of the women had lower to middle responsibility and skill jobs and 49.8% reported a family income below 2,000 Euros a month. Income trends are U-shaped with the highest percentages of low earners among the first and last age stratum, i.e., below 35 and above 65 years of age. About one third of the women reported living alone, with a significant increase with increasing age. Additionally, 70.2% of the women stated that they ever had children, with a prevalence of 86.6% in the oldest age stratum. In accordance with the structure of the German health care system, no women reported absence of any form of health insurance; 68% had only statutory insurance coverage, with an increasing trend with age. Most women had a general practitioner and 69 to 78% reported attending their office at least once during the 3 months prior to the study.

**Table 1:** **Sociodemographic information and risk factor distribution**

	**Total (n = 1,062)**	**Stratum 1 (25–34) n = 203**	**Stratum 2 (35–44) n = 205**	**Stratum 3 (45–54) n = 220**	**Stratum 4 (55–64) n = 211**	**Stratum 5 (65–75) n = 223**
**Sociodemography**						
Age (mean)	50.3 (25–75)	30 (25–34)	40.3 (35–44)	49.7 (45–54)	59.4 (55–64)	70 (65–75)
Education (≤10 years)	490 (46.1%)	49 (24.1%)	87 (42.4%)	104 (47.3%)	110 (52.1%)	140 (62.8%)
Profession (low status)	623 (65.9%)	92 (65.9%)	120 (66.3%)	133 (64.9%)	138 (70.8%)	141 (70.9%)
Income (<2,000 Euros/month)	464 (49.8%)	106 (57.3%)	70 (39.1%)	88 (43.6%)	80 (44.4%)	120 (64.5%)
Family situation (living alone)	332 (31.3%)	63 (31%)	36 (17.6%)	55 (25%)	83 (39.3%)	95 (42.6%)
Parity (any no. of children)	746 (70.2%)	61 (30%)	144 (70.2%)	176 (80%)	172 (81.5%)	193 (86.6%)
Not working^a^	341 (32.11%)	31 (15.27%)	34 (16.6%)	26 (11.82%)	66 (31.3%)	184 (82.5%)
**Health care**						
Insurance status (public only)	151 (68%)	158 (78.6%)	141 (68.8%)	149 (68%)	136 (65%)	151 (68%)
Availability of GP (no GP)	155 (14.6%)	41 (20.2%)	35 (17.2%)	37 (16.9%)	30 (14.2%)	12 (5.4%)
Any CV medication	279 (26.27%)	7 (3.45%)	16 (7.8%)	51 (23.18%)	81 (38.39%)	124 (55.61%)
Any medical consultation within last 3 months	774 (73.2%)	141 (69.5%)	141 (69.1%)	162 (74%)	155 (73.8%)	175 (78.8%)
**Risk factors**						
Smoking (active)	296 (28%)	72 (35.6%)	77 (37.6%)	68 (31.3%)	57 (27.1%)	22 (9.9%)
Diabetes mellitus	55 (5.2%)	0	7 (3.4%)	8 (3.7%)	11 (5.2%)	29 (13.2%)
Hypertension	281 (26.5%)	8 (4%)	18 (8.8%)	53 (24.2%)	80 (37.9%)	122 (54.7%)
Obesity (BMI >30)	168 (15.8%)	11 (5.4%)	28 (13.7%)	35 (15.9%)	43 (20.4%)	51 (22.9%)
(WHR >0.86)	265 (25%)	10 (4.9%)	38 (18.54%)	64 (29.1%)	64 (30.3%)	89 (39.9%)
Hyperlipidemia (Tot/HDL ratio)	142 (13.4%)	10 (4.9%)	31 (15.1%)	30 (13.6%)	40 (19%)	31 (13.9%)
Any family history (positive)	851 (80.3%)	164 (80.8%)	167 (81.5%)	184 (83.6%)	176 (83.4%)	160 (71.8%)
Previous CV event	27 (2.5%)	2 (1%)	1 (0.5%)	3 (1.4%)	8 (3.8%)	13 (5.8%)
Atrial fibrillation	3 (0.28%)	0	0	1 (0.45%)	0	2 (0.9%)
Autoimmune disease	125 (11.9%)	10 (4.95%)	22 (10.84%)	21 (9.68)	37 (17.87)	35 (15.84%)
Postmenopausal	534 (51.4%)	5 (2.5%)	20 (9.8%)	94 (43.12%)	206 (99.04%)	209 (100%)
Pregnancy complications	87 (8.19%)	6 (2.96%)	16 (7.8%)	15 (6.82%)	25 (11.85%)	25 (11.21%)
Depressive mood^b^ (PHQ9 ≥ 15)	52 (5.2%)	10 (5.1%)	13 (6.63%)	14 (6.86%)	11 (5.47%)	4 (1.97%)
(PHQ9 ≥ 10)	168 (16.8%)	42 (21.4%)	34 (17.4%)	36 (17.6%)	32 (15.9%)	24 (11.8%)

^a^Unemployment or retirement; ^b^Both cutoffs were reported to allow for comparison with national and international surveys.

CV, Cardiovascular; HDL, High-density lipoprotein; WHR, Waist-to-hip ratio; PHQ9, Patient health questionnaire.

### Risk factor distribution with age

We assessed common and female-specific cardiovascular risk factor prevalence with age (Table 1). As expected, active smoking prevalence decreased with age while diabetes, hypertension, hyperlipidemia, obesity, and vascular compliance measured by pulse pressure values demonstrated a linear increase with age. While none of the women below 34 years of age suffered from diabetes, 13.2% above 65 years of age did. Hypertension affected more than 50% of the women in the highest age stratum. Obesity was assessed with two different criteria, body mass index (BMI) and waist-to-hip ratio (WHR), to account for the increased pathologic relevance of abdominal obesity. In fact, while only 23% of the women above 65 years were classified as obese according to BMI, 40% displayed abdominal obesity. Interestingly, women below 34 years displayed a lower incidence of obesity according to WHR compared to BMI measurement, while in all other age groups diagnosis according to WHR proved more stringent. Pregnancy complications were reported slightly more frequently with increasing age. The prevalence of depressive symptoms (Patient Health Questionnaire (PHQ9) ≥10) displayed a constant prevalence between 16 and 21% throughout all age groups, but the highest.

### Subjective estimation of risk

To determine correctness of self-estimation of risk, we compared subjectively asserted cardiovascular risk on a 3-point Likert scale with the objective risk measured according to the Framingham risk score for women [21,24]. In our population, only 41.35% of all the participants correctly identified their own risk, whereas 48.65% underestimated it and 10% overestimated it (Table 2). In univariate analysis, age appeared as the single most predictive factor for underestimation with women in the highest age group 13.8 times more likely to underestimate their risk compared to the youngest. In univariate analyses, several biological risk factors were associated with a trend for underestimation of risk (Additional file 2: Table S2); nevertheless, after adjustment for age and other covariates in the multivariate analysis, none of these associations was confirmed due to the strong collinearity with age. Age and social risk factors, however, proved significant even after adjustment for all possible confounders. Moreover, age was confirmed as the single most significant predictor of underestimation with women over 50 years of age 3.5 times more likely to underestimate their risk (odds ratio (OR) = 3.5, 95% confidence interval (CI) = 2.6–4.8, *P* <0.0001; Tables 3 and 4). Joblessness, either due to unemployment or retirement, also increased the risk of underestimation by almost 2 times. Social risk factors were, however, most powerful in combination. In fact, while low income, limited education, a simple job, living alone, having children, and having only statutory coverage were not associated with underestimation taken singularly, the unfavorable combination of three or more of these factors increased the risk of underestimation by 1.5 times.

**Table 2:** **Subjective estimation vs. Framingham score (stratified according to D’Agostino** [24]**)**

	**High Framingham score**	**Medium Framingham score**	**Low Framingham score**
**Risk estimate high**	4.49%	4.69%	0.5%
	(n = 45)	(n = 47)	(n = 5)
**Risk estimate medium**	16.85%	25.42%	4.79%
	(n = 169)	(n = 255)	(n = 48)
**Risk estimate low**	6.78%	25.02	11.47%
	(n = 68)	(n = 251)	(n = 115)

**Table 3:** **Multiple regression analysis of predictors for subjective under- and overestimation of cardiovascular risk: underestimation**

	**OR**	**95% CI**	***P***
Age >50 years	3.5	2.6–4.8	<0.0001
Joblessness	1.9	1.4 –2.6	<0.0001
Social risk factors (3 or more)	1.5	1.1–2.1	0.009
Positive subjective health rating	1.7	1.2–2.3	0.003

Goodness-of-fit (Wald) n.s.; Hosmer-Lemeshow n.s.; ROC for prediction 0.71.

**Table 4:** **Multiple regression analysis of predictors for subjective under- and overestimation of cardiovascular risk: overestimation**

	**OR**	**95% CI**	***P***
Age >50 years	0.45	0.24–0.87	0.02
Negative Subjective Health Rating	2.7	1.4–5.1	0.002
Depression (PHQ ≥10)	2.2	1.2–4.0	0.014
Low income	0.48	0.28–0.84	0.009
Simple/Mid-level Job	0.59	0.41–0.83	0.003

Goodness-of-fit (Wald) n.s.; Hosmer-Lemeshow n.s.; ROC for prediction 0.75.

As expected, older age was negatively correlated with overestimation (OR = 0.45, 95% CI = 0.24–0.87, *P* = 0.02, Tables 3 and 4), as were low income and simple to mid-level employment. Subjective negative rating of the overall health status was a significant predictor of overestimation of risk (OR = 2.7, 95% CI = 1.4–5.1, *P* = 0.002), as were depressive symptoms (PHQ ≥10; OR = 2.2, 95% CI = 1.2–4.0, *P* = 0.014). However, while 40.7% of all overestimators reported symptoms compatible with major depression, only 22.9% of the depressed participants actually overestimated their risk.

### Lack of awareness of objective risk factors

Next to subjective awareness of cardiovascular risk, we also evaluated the objective knowledge about risk factors in the participant population. Women were asked whether they knew optimal values of blood pressure, fasting glucose, and cholesterol using multiple-choice questions and to rate the importance of age as a risk factor on a range from 1 to 10. All of these parameters displayed trends for modification with increasing age. Knowledge of ideal values of blood pressure (*P* <0.0001), cholesterol (*P* <0.0001), and fasting glucose (*P* = 0.03) all increased with age, probably due to an increase in relevance. In fact, among the population of diabetics, 59.6% knew the ideal values of fasting glucose, while only 27.8% of the non-diabetics were aware of these numbers (*P* <0.0001). The same applied for hypertension (88% among known hypertensives vs. 79.6% in normotensives, *P* = 0.002) and hyperlipidemia (36.6% among hyperlipidemics vs. 26.7% non-hyperlipidemics, *P* = 0.03). As expected, job experience in the health care sector significantly correlated with correct estimation of ideal blood pressure, fasting glucose, and cholesterol (all *P* <0.0001), yet it did not impact the perceived relevance of age as an important risk factor (63.2% among health care experienced individuals vs. 61.9% among the general population, *P* = 0.69). Age was rated an important risk factor by 62.1% of the study population, yet this perception only weakly increased with increasing age (*P* = 0.046). Overall, knowledge about objective risk parameters did not significantly associate with correctness of estimation.

## Discussion

We designed this study to evaluate women’s estimation of their subjective cardiovascular risk, as this appears to be one of the potential causal factors on the path from underestimation of cardiovascular relevance to cardiovascular mortality. To our knowledge, the BEFRI study was the first attempt to elucidate these factors. First, we could confirm that both typical cardiovascular risk factors as well as female-specific risk factors correlate with age. Second, we discovered that subjective underestimation of cardiovascular risk significantly rises with age and the presence of three or more unfavorable social risk factors. Third, we determined how consciousness of age as a cardiovascular risk factor only minimally increases with the age of the rating subject. Overall, this reveals how women at increased risk are also the ones less aware of their risk and, thus, identifies new challenges in the development of future preventative measures.

Increasing age has been described as a cardiovascular risk factor from the start of cardiovascular epidemiology [25]. The prevalence of atherosclerosis [26], metabolic dysfunction [27], and immune dysregulation [28] all increase with age, resulting in an increased incidence of hypertension, diabetes, dyslipidemia, and vascular dysfunction [29]. These phenomena have been described in both sexes, although the relevance of these risk factors is more prominent in women compared to men [30]. The attention has only recently shifted to the role of ‘female-specific’ risk factors, such as pregnancy complications, autoimmune diseases, and increased incidence of depressive and mood disorders, as potential predisposing risks for cardiovascular events [21,31], leading to the inclusion of gender and ethnicity as segregating factors for the novel ACC/AHA risk estimator, which also includes the risk of experiencing a stroke [32]. Furthermore, recent reports of a potentially biphasic effect of estrogens, not just as protectors against cardiovascular risk factors, but also as potential accelerators under certain circumstances, should be considered [33]. In our study, we systematically assessed all these aspects in a representative sample of an urban German population and confirmed that classic risk factors linearly associate with age – rather than precipitously increasing after menopause – and that this also applies to most female-specific risk factors, although to a less pronounced degree. In fact, pregnancy complications experienced by the participants did increase with age, yet the differences among the highest age band and the lowest were about 10%. While this might be due to a reporting bias, it also confirms a physiological decrease due to improvement of care over the last decades in a population that had acceptable standards to begin with. Autoimmune diseases display two hormone-associated incidence peaks, during the fertile period of a woman’s life and after menopause, yet different diseases display different associations with hormonal status [34,35] and this might well explain a progressive yet contained surge over time. However, one should also consider that being a population study and not a hospital-based investigation, a ‘healthy participant’ bias might prevent women with significant disability to participate in the study. This will have inevitably excluded some individuals from being captured with this sampling strategy, albeit equally at all ages. The same probably also applies to participants with depression. Prevalence of depression does not vary significantly in the different age groups below 65 years of age, which is well in line with recent demography studies of the German population [36]. In the study population over 65 years of age, however, prevalence numbers decreased significantly. Since a real reduction in this age group appears unlikely, this could be explained either by the increased mortality in depressed patients, given the association of depression with morbidity [37,38], or the presence of other co-morbidities in association with depression that impeded participation in the study.

Although to physicians age is a well-documented risk factor for any disease, the perception of this aspect in the general female population and the consequent adaptation of one’s subjective risk perception do not appear to follow. Interpretation of the concept of subjective risk might also have been skewed, yet, this phenomenon has been described to various ends in the literature. In fact, both the association between risk factors and perception [39] and absence thereof have been described [40]. Some authors also identified a variance in risk perception depending on whether the question was asked in absolute or comparative – to a group of peers – terms [41]. Interestingly, in our population the older the women the more likely they appeared to underestimate their subjective risk. However, this was not only a subjective underestimation, which has been described before as ‘unrealistic optimism’ [42], but an invariant perception of the role of age over time. Since cardiovascular risk increases with age and comorbidities over time, and this is reflected substantially in risk scores, the simple unawareness of this phenomenon will lead to a significant misclassification of one’s own risk. In addition to the role of age, social risk factors appeared as major predictors of underestimation.

Joblessness, due to either unemployment of retirement, significantly coincided with underestimation – irrespective of age. Most likely, the psychosocial benefits of work life [43] affect both risk development and ability to estimate subjective risk, possibly due to peer comparison. Absence of the routine and social integration that work life provides, directly affected risk perception. Other social risk factors, such as limited education, means, lower job opportunities, living alone, having children, and absence of integrative insurance coverage, were associated with underestimation only if combined in sets of three or more. This might be well explained by the functioning welfare state in Germany, which appears effective in subsidizing single social inequities, and by the compensatory function of some factors towards others. For instance, having children is not a social risk factor if the subject is well educated and has a profitable job, yet, it does constitute an additional risk factor if the individual is poorly educated and earns a meager income.

Some aspects should, nonetheless, be taken into consideration. The following study is a good representation of the urban population of Berlin, yet it is probably much less representative of rural areas in Germany or some other parts of Europe. Even with little education, the exposure to information within a large city differs significantly from rural areas, leading to different flows of information and possibly also disinformation. Women have been asked to voluntarily participate in the study, introducing a selection bias, which might have been reinforced by the payment of an incentive. However, the population is a good representation of the city as whole and the payment of an incentive was only communicated after consent to participation had been given, thus both these aspects have been minimized. Furthermore, awareness and knowledge have been asserted by a questionnaire and not by direct interviewing, allowing for a potential bias in education and concentration for the duration of the process. This was minimized through an extensive piloting phase, where questionnaires were evaluated with a heterogeneous population, and has been confirmed by the very limited rate of missing answers to the questionnaire. Finally, we chose the Framingham global cardiovascular risk score for women, which might overestimate risks as described in the past; while this was confirmed for the Framingham risk score for coronary heart disease [44], it has not been described for the global cardiovascular risk score. Nonetheless, the possibility of overestimation has to be taken into account and could potentially alter the relationship between subjective perception and objective measure, i.e., increase the reported underestimation of risk. Every score has inherent advantages and disadvantages and comparisons are oftentimes difficult [45]; thus, we chose the most validated one and the one specifically proposed in the guidelines for cardiovascular prevention in women [21].

## Conclusions

The present study is the first evaluation of women’s subjective risk estimation demonstrating how the most vulnerable individuals, the elderly, and the socially disadvantaged bear the greatest risk of subjective underestimation. Since the acceptance and uptake of preventive measures is inherently linked to the self-perception of relevance, this result leads us to conclude that much needs to be done to improve our population’s health literacy and risk awareness. It appears that, despite the relevance of age being well known, the transfer of this concept to one’s self is problematic. We urge that this aspect be emphasized in risk counselling in primary and specialist care, but also be addressed through targeted information campaigns.

## Additional files

Additional file 1: Table S1. Comparison general female population of the city of Berlin – BEFRI study population. Additional file 2: Table S2. Univariate association analyses.

## Abbreviations

BEFRI: Berlin Female Risk Evaluation
BMI: Body mass index
CI: Confidence interval
ECG: electrocardiogram
OR: Odds ratio
PHQ: Patient health questionnaire
WHR: Waist-to-hip ratio
