# Supplementary material for: Cardiovascular risk factor distribution and subjective risk estimation in urban women – The BEFRI Study: a randomized cross-sectional study
Source: BMC Med. 2015 Mar 16;13:52. doi: 10.1186/s12916-015-0304-9 (PMC4373038; doi:10.1186/s12916-015-0304-9)
Supplement: Additional file 1: Table S1. — Comparison general female population of the city of Berlin – BEFRI study population. [file 12916_2015_304_MOESM1_ESM.doc]

Supplementary Table 1.

Comparison general female population of the city of Berlin – BEFRI study population

A) Demography and social factors

|  | **General population*** | **BEFRI study** |
| --- | --- | --- |
| Education (low) | 50.3% | 46.1% |
| Professional Status (low) | 56.9% | 65.9% |
| Income (< 2000 Euros/mo)^a^ | 61.5% | 49.8% |
| Living alone | 36.4% | 31.3% |
| Parity^b^ | NA | 70.2% |
| Joblessness^c^ | 29.2% | 32.11% |

* general population data from statistics department of Berlin-Brandenburg (data in this group is not differentiated for migration background in all analyses; statistics are collected for the population 15-65 years of age)

^a^ collected on an individual basis by the census vs family unit basis in BEFRI

^b^ information about parity is not collected on a lifetime basis by the Berlin census

^c^ data from Microcensus, age range 25 to 70 years

B) General risk factors

|  | **Age 45-64*** | **BEFRI 45-64**^a^ | **Age > 65*** | **BEFRI 65 – 74**^a^ |
| --- | --- | --- | --- | --- |
| Smoking (active) | 29.4 % | 29.2 % | 8.7 % | 9.9 % |
| Diabetes mellitus | 7.7 % | 4.45 % | 20.1 % | 13.2 % |
| Hypertension | 33.3 % | 31.05 % | 57.8 % | 54.7 % |
| Obesity (BMI >30) | 18.8 % | 18.15 % | 22 % | 22.9 % |

* Modified and reproduced with permission from Lange C, Ziese T, “Daten und Fakten: Ergebnisse der Studie “Gesundheit in Deutschland aktuell 2009““, Robert Koch Institut, Berlin, 2011

^a^ mean of stratum 45-64
