# Supplementary material for: Cardiovascular risk factor distribution and subjective risk estimation in urban women – The BEFRI Study: a randomized cross-sectional study
Source: BMC Med. 2015 Mar 16;13:52. doi: 10.1186/s12916-015-0304-9 (PMC4373038; doi:10.1186/s12916-015-0304-9)
Supplement: Additional file 2: Table S2. — Univariate association analyses. [file 12916_2015_304_MOESM2_ESM.doc]

Supplementary Table 2 – Univariate Association Analyses

|  | **Underestimation** | **Correct Estimate** | **p** | **Overestimation** | **Correct Estimate** | **p** |
| --- | --- | --- | --- | --- | --- | --- |
| **Sociodemography** |  |  |  |  |  |  |
| Age (>50.3 years) | 68.65% | 35.18% | < 0.0001 | 19.00% | 35.18% | 0.002 |
| Education (low) | 51.43% | 38.31% | < 0.0001 | 39% | 38.31% | n.s. |
| Profession (low) | 72.77% | 62.75% | 0.003 | 58.9% | 62.75% | n.s. |
| Biomedical Profession | 81.78% | 82.44% | n.s. | 80% | 82.44% | n.s. |
| Income | 50.36% | 54.93% | n.s. | 34.44% | 54.93% | < 0.0001 |
| Family Situation | 31.35% | 29.40% | n.s. | 34% | 29.4% | n.s. |
| Parity | 77.24% | 63.61% | < 0.0001 | 56% | 63.61% | n.s. |
| Insurance Status | 66.19% | 69.64% | n.s. | 79% | 69.64% | n.s. |
| Non-availability of GP | 12.7% | 17.19% | 0.059 | 16% | 17.19% | n.s. |
| Medical consultation (last 3 mo) | 72.43% | 73.43% | n.s. | 73% | 73.43% | n.s. |
| Any CV medication | 20.48% | 30.74% | < 0.0001 | 22% | 20.48% | n.s. |
|  |  |  |  |  |  |  |
| **Risk Factors** |  |  |  |  |  |  |
| Smoking | 26.64% | 26.51% | n.s. | 66% | 73.49% | n.s. |
| Diabetes Mellitus | 7.38% | 3.61% | 0.015 | 2% | 3.61% | n.s. |
| Hypertension | 30.33% | 21.45% | 0.003 | 23.71% | 21.45% | n.s. |
| Obesity | 17.83% | 12.53% | 0.028 | 18% | 12.53% | n.s. |
| Hyperlipidemia | 15.78% | 10.36% | 0.017 | 7% | 10.36% | n.s. |
| Family history | 77.05% | 82.17% | 0.058 | 87% | 82.17% | n.s. |
| Previous CV Event | 2.66% | 2.66% | n.s. | 2% | 2.66% | n.s. |
| Atrial fibrillation | 0 | 100% | < 0.0001 | 100% | 0 | < 0.0001 |
| Autoimmune Disease | 12.99% | 10.78% | n.s. | 10% | 10.78% | n.s. |
| Peri/Postmenopausal | 68.97% | 36.79% | < 0.0001 | 24.24% | 36.79% | 0.018 |
| Pregnancy Complications | 9.22% | 6.99% | n.s. | 7% | 6.99% | n.s. |
